# Impact of borderline pulmonary hypertension due to left heart failure on mortality in a multicenter registry study: A 3-year survivorship analysis

**DOI:** 10.3389/fcvm.2022.983803

**Published:** 2022-08-12

**Authors:** Yangyi Lin, Lingpin Pang, Shian Huang, Jieyan Shen, Weifeng Wu, Fangming Tang, Weiqing Su, Xiulong Zhu, Jingzhi Sun, Ruilin Quan, Tao Yang, Huijun Han, Jianguo He

**Affiliations:** ^1^Department of Pulmonary Vascular Disease, State Key Laboratory of Cardiovascular Disease, Fuwai Hospital, National Center for Cardiovascular Diseases, Chinese Academy of Medical Sciences & Peking Union Medical College, Beijing, China; ^2^Cardiovascular Medicine Center, Affiliated Hospital of Guangdong Medical University, Zhanjiang, China; ^3^Department of Cardiology, Renji Hospital, Shanghai Jiaotong University School of Medicine, Shanghai, China; ^4^Department of Cardiology, The First Affiliated Hospital of Guangxi Medical University, Nanning, China; ^5^Department of Cardiology, Nongken Central Hospital of Guangdong Province, Zhanjiang, China; ^6^Department of Cardiology, Lianjiang People's Hospital, Lianjiang, China; ^7^Department of Cardiology, People's Hospital of Gaozhou, Gaozhou, China; ^8^Department of Cardiology, Affiliated Hospital of Jining Medical University, Jining, China; ^9^Department of Epidemiology and Biostatistics, Institute of Basic Medical Sciences, Chinese Academy of Medical Sciences and School of Basic Medicine, Peking Union Medical College, Beijing, China

**Keywords:** borderline pulmonary hypertension, left heart failure, mean pulmonary artery pressure (mPAP), mortality, right heart catheterization (RHC)

## Abstract

**Background:**

Patients with left heart failure (LHF) are often associated with the development of pulmonary hypertension (PH) which leads to an increased risk of death. Recently, the diagnostic standard for PH has changed from mean pulmonary arterial pressure (mPAP) ≥25 mmHg to >20 mmHg. Nonetheless, the effect of borderline PH (mPAP: 21–24 mmHg) on the prognosis of LHF patients is unclear. This study aimed to investigate the relationship between borderline PH and 3-year clinical outcomes in LHF patients.

**Methods:**

A retrospective analysis of a prospective cohort study was done for LHF patients who underwent right heart catheterization (RHC) between January 2013 and November 2016. The primary outcome was all-cause mortality; the secondary outcome was rehospitalization.

**Results:**

Among 344 patients, 62.5% were identified with a proportion of PH (mPAP ≥ 25), 10.8% with borderline PH (21–24), and 26.7% with non-PH (≤20), respectively. Multivariable Cox analysis revealed that borderline PH patients had a higher adjusted mortality risk (HR = 3.822; 95% CI: 1.043–13.999; *p* = 0.043) than non-PH patients. When mPAP was treated as a continuous variable, the hazard ratio for death increased progressively with increasing mPAP starting at 20 mmHg (HR = 1.006; 95% CI: 1.001–1.012). There was no statistically significant difference in adjusted rehospitalization between borderline PH and non-PH patients (HR = 1.599; 95% CI: 0.833–3.067; *p* = 0.158).

**Conclusions:**

Borderline PH is independently related to increased 3-year mortality in LHF patients. Future research is needed to evaluate whether more close monitoring, and managing with an intensifier improves clinical outcomes in borderline PH caused by LHF.

**Clinical trials registration:**

www.clinicaltrials.gov NCT02164526.

## Introduction

Pulmonary hypertension (PH) due to left heart failure (PH-LHF), also known as post-capillary pulmonary hypertension [pulmonary arterial wedge pressure (PAWP) >15 mmHg], is the most prevalent kind of PH, affecting around 5% of people aged 65 and older (1). Elevated mean pulmonary arterial pressure (mPAP) of ≥25 mmHg determined by right heart catheterization (RHC) at rest in supine position is the essential condition for PH diagnosis (2, 3). However, in the 6th World Symposium on Pulmonary Hypertension (WSPH), this threshold value was dropped to 20 mmHg to define PH for all subgroups (mPAP > 20 mmHg) (4). Nonetheless, data on modestly raised mPAP (21–24 mmHg), sometimes known as borderline PH, remain scarce (5). This proposal has sparked extensive debate among academic institutions (6, 7).

Opponents have stated that the diagnosis is “life-threatening,” but no approved or evidence-based therapy is available so far, the immediate and profound psychological damage may outweigh the benefits of an early PH diagnosis. Furthermore, physicians may face treatment dilemmas, such as whether borderline PH patients may be administered off-label treatment (6). As no specific PH treatment is currently available, the new criterion has little or no impact on therapy for PH-LHF patients (8). Should the new hemodynamic criterion be worth adopting in LHF patients? Therefore, investigating the outcome of borderline PH-LHF will provide an essential foundation for deciding whether to adopt the new criterion or not.

Although previous researches have suggested that mPAP may be prognostic in patients with left heart disease, it is unknown whether borderline PH worsens mortality in people with LHF. For instance, one study revealed that mPAP is the strongest hemodynamic predictor of mortality in patients with LHF. However, this study did not analyze whether there is a survival difference between patients without PH (mPAP ≤ 20 mmHg) and those with borderline PH (9). Additionally, two large cohort studies demonstrated that the borderline PH is associated with an increased risk of death (10, 11). Nevertheless, they defined borderline PH as mPAP between 19 and 24 mmHg, and LHF proportion was 8.8 and 48.2%, respectively, in their recruited patients. As a result, their outcomes may be insufficient in LHF patients. Thus, our study sought to determine whether borderline PH is related to higher mortality in LHF patients.

## Materials and methods

### Study design and participants

**Figure 1:**
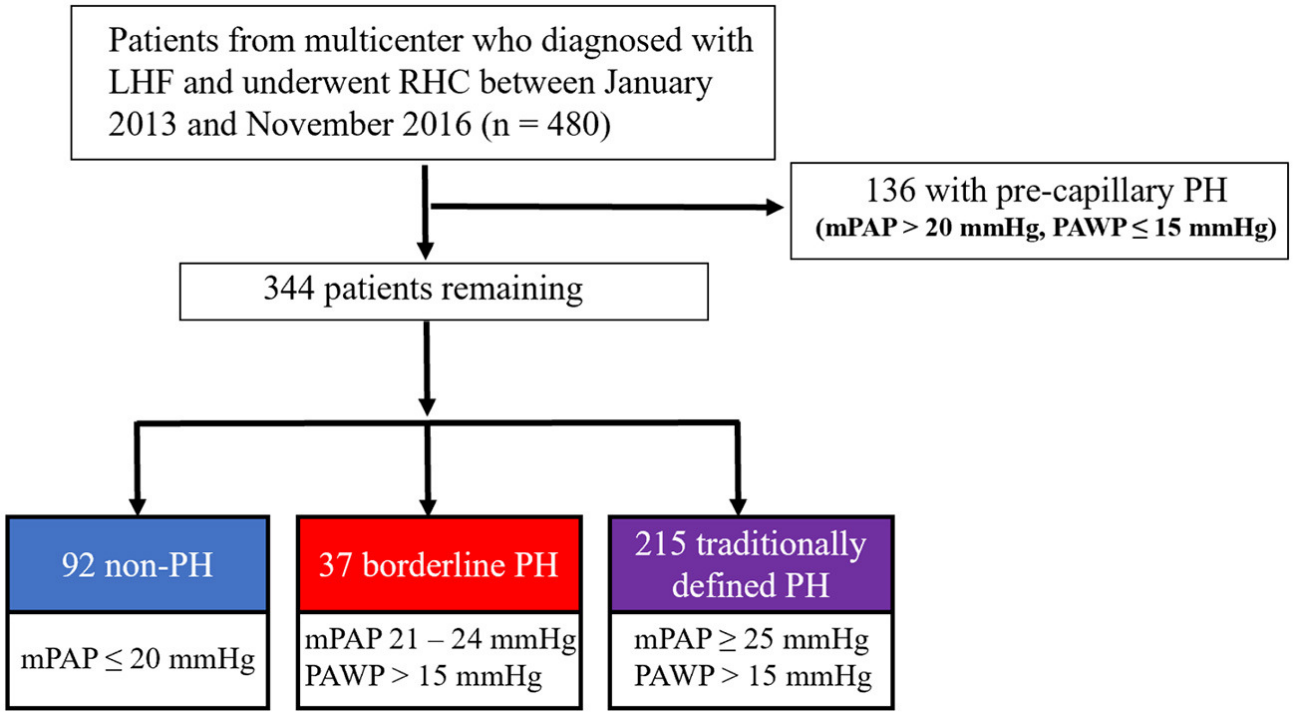
The study design and flowchart for the selection of patients. LHF, left heart failure; RHC, right heart catheterization; PH, pulmonary hypertension.

The study design and patient selection flowchart is shown in Figure 1, which was a retrospective analysis of a prospective, multicenter registry study of LHF patients who received RHC between January 2013 and November 2016. The study protocol was approved by Fuwai Hospital's Institutional Review Board (Approval No. 2012-401) and was carried out adopting the Helsinki Declaration, and was registered on ClinicalTrials.gov (Identifier: NCT02164526). All patients enrolled were provided written informed consent.

Patients were enrolled in the study according to the following criteria: (1) patients with a verified diagnosis of LHF following the current heart failure guideline (12). (2) patients who underwent RHC between January 2013 and November 2016. Patients were excluded if they met any of the following criteria: (1) hypertrophic obstructive cardiomyopathy; (2) right ventricular outflow tract stenosis; (3) pericardial disease; (4) patients with chronic lung disease; (5) HF due to valvular heart disease; (6) pre-capillary PH (mPAP > 20 mmHg, PAWP ≤ 15 mmHg).

### Measurements and data collection

PH-LHF patients were extensively clinically assessed by PH experts to exclude PH due to other etiologies. Biochemical blood tests were performed within 24-h of admission. The initial measurement on admission was used to acquire the blood pressure, heart rate, echocardiographic, and biochemical parameters. RHC and left heart catheterization were used to achieve hemodynamic parameters. RHC was conducted to confirm a physician's diagnosis of suspected PH-LHF, performed in stable and non-acute clinical settings. PAWP was measured at end-diastole at rest; when PAWP measurement is unreliable, left cardiac catheterization was used to determine left ventricular end-diastolic pressure (LVEDP). Traditionally defined PH is an increase in mPAP ≥ 25 mmHg at rest; borderline PH is defined as an mPAP value of 21–24 mmHg. Coronary artery disease (CAD) is defined as 50% or more stenosis of at least one coronary artery by quantitative coronary angiography or having a prior physician-documented history of CAD. HFpEF, HFmrEF, and HFrEF are defined as left ventricular ejection fraction (LVEF) ≥ 50%, LVEF 41–49%, and LVEF ≤ 40%, respectively. All enrolled patients had data from two-dimensional echocardiography and RHC. Medical histories, demographics, baseline clinical and radiograph data, laboratory results, and treatments were reviewed from our registry study's database records.

### Exposure

The exposure was mPAP as reported in our dataset. To determine if borderline PH affects mortality in patients with LHF, patients were divided into three groups i.e., non-PH (mPAP ≤ 20 mmHg), borderline PH (mPAP: 21–24 mmHg), and the traditionally defined PH (mPAP ≥ 25 mmHg) (2).

### Outcomes and follow-up

Our primary outcome measure was the time interval between enrolment and all-cause mortality. The secondary outcome measure was the time interval between enrollment and rehospitalization for any reason. Every 6 months ± 2 weeks, patients were followed up by phone calls, messages, or outpatient visits, and it was confirmed whether they died or were re-hospitalized at each follow-up. Patients who could not be reached by phone, message, hospital system, or other available means more than three times and lasted for more than 6 months were defined as lost to follow-up.

### Missing and extreme data

Linear interpolation was used to handle the missing variables, which were then fed into the multivariable model for analysis. Missing data was defined as the absence of both values concurrently for variables with the same clinical significance, such as BNP and NT-proBNP. Biomarker levels below the detection limit were set to half that level, while those over the detection limit were set to the upper limit level. Hemodynamic parameters were examined for physiologically incredible values, which were classified as mPAP <5 or >80 mmHg, and PAWP <0 or >60 mmHg. As a result, none of the patients possessed extraordinary values.

### Statistical analysis

The statistical analysis was performed using R (version 4.0.2) and SPSS (version 24.0). Continuous variables were presented as mean ± standard deviation for normally distributed data, or, in case of skewed distributions, median with interquartile range (IQR, 25th−75th percentiles), categorical variables were reported as counts and percentages (%). Whenever appropriate, continuous variables were transformed into categorical variables using a median or mean in regression analysis. The baseline demographic, clinical, and hemodynamic characteristics of non-PH, borderline PH, and traditionally defined PH groups were compared using one-way analysis of variance (ANOVA) with least significant difference (LSD) *post hoc* test or Games-Howell *post hoc* test for normally distributed variables, Kruskal-Wallis with Bonferroni correction *post hoc* test for skew distributed variables and the Chi-square test for categorical variables. We employed one-way ANOVA with trend analysis or Linear-by-Linear Associated trend analysis to determine whether variables tended in one direction across groups. To identify factors linked with PH, univariate and multivariate logistic regression were used. Kaplan-Meier survival curves were produced using either death or rehospitalization as events, and log-rank tests were used to make unadjusted group comparisons for time to event outcomes. Patients with more than 3 years of follow-up were censored after 36 months. Univariate Cox proportional hazard regression analyses were followed by multivariate Cox proportional hazard regression to identify predictors of death or rehospitalization. The proportionality of hazards for each variable was determined by examining the statistical significance of interactions between follow-up time and variables. A cubic spline model was built to describe the association between mPAP and all-cause mortality hazard ratio (HR); the number of knots was chosen to produce the best fit as measured by the Akaike information criteria. To determine the predictive accuracy of mPAP for mortality, the Youden's index and area under the curve of the time-dependent receiver operating characteristic (ROC) curve were determined. The statistical significance level was established at 0.05 on a two-sided scale.

## Results

### Characteristics of study population

After applying the inclusion and exclusion criteria, 92 (26.7%) patients were found to be without PH, 37 (10.8%) with borderline PH, and 215 (62.5%) with traditionally defined PH and were regarded as appropriate for this study (Figure 1). This cohort study patients were primarily male subjects (*n* = 253, 73.5%) and had been diagnosed with LHF within 30 days (*n* = 298, 86.6%) with a mean age of 63.3 years (standard deviation [*SD*], 12.0 years) for all included patients. The median mPAP was 27 mmHg (interquartile range [IQR], 20–32 mmHg; minimum, 7 mmHg; maximum, 70 mmHg), and the distribution of mPAP is depicted in the histogram (Supplementary figure S1). The median PAWP was found to be 19 mmHg (IQR, 17–24 mmHg; minimum, 16 mmHg; maximum, 45 mmHg), and mean ejection fraction (EF) was 54.2% (*SD*, 10.6%; minimum, 16%; maximum, 80.0%). CAD was the most prevalent co-morbidity (*n* = 263, 76.5%); among which, 208 (79.1%) had HFpEF, 32 (12.2%) had HFmrEF, and 23 (8.7%) had HFrEF. Table 1 summarizes the cohort's baseline demographic, clinical, and hemodynamic parameters. Except for the uric acid and hemodynamic variables determined by RHC, there were no significant differences between the non-PH and borderline PH groups (Table 1). Covariate variables had missing values ranging from 0.3% for platelets to 12.2% for natriuretic peptides (Table 1).

**Table 1:** Baseline demographic, clinical and hemodynamic characteristics by non-PH, borderline PH, and traditionally defined PH status.

	**mPAP (mmHg)**				
	**≤20 (*n* = 92)**	**21–24 (*n* = 37)**	**≥25 (*n* = 215)**	**P value**	***P* for trend**
Age (years)	62.7 ± 11.1	61.8 ± 11.8	63.8 ± 12.4	0.557	0.401
Female, *n* (%)	19 (20.7%)	9 (24.3%)	63 (29.3%)	0.317	0.498
BMI (kg/m^2^)	22.3 ± 2.6	21.8 ± 2.0	23.2 ± 3.0^*#^	0.005	0.006
**NYHA FC**, ***n*** **(%)**					
II	72 (78.3%)	30 (81.1%)	126 (58.6%)^*#^	0.001	<0.001
III/IV	20 (21.7%)	7 (18.9%)	89 (41.4%)^*#^	0.001	<0.001
**Types of HF**					
HFpEF, *n* (%)	76 (82.6%)	31 (83.8%)	148 (68.8%^*#^	0.015	0.007
HFmrEF, *n* (%) ^∫∫^	3 (3.3%)	3 (8.1%)	40 (18.6%)		
HFrEF, *n* (%) ^∫∫^	13 (14.1%)	3 (8.1%)	27 (12.6%)		
Heart rate (bpm)	74.1 ± 14.6	75.7 ± 12.9	76.8 ± 15.2	0.327	0.136
Respiratory rate (bpm)	19.3 ± 1.8	18.9 ± 2.2	19.4 ± 1.9	0.268	0.425
SBP (mmHg)	138.1 ± 23.2	135.7 ± 20.0	132.7 ± 22.8	0.152	0.052
DBP (mmHg)	79.5 ± 14.5	75.4 ± 12.1	76.3 ± 12.1	0.100	0.064
CAD, *n* (%)	77 (83.7%)	30 (81.1%)	156 (72.6%)	0.085	0.029
Hypertension, *n* (%)	41 (44.6%)	18 (48.6%)	100 (46.5%)	0.906	0.791
Hyperlipidemia, *n* (%)	20 (21.7%)	7 (18.9%)	60 (27.9%)	0.335	0.208
Diabetes, *n* (%)	25 (27.2%)	8 (21.6%)	59 (27.4%)	0.757	0.878
Ischemic stroke, *n* (%) ^∫∫^	7 (7.6%)	2 (5.4%)	16 (7.5%)		
**Biochemistry**					
Hemoglobin (g/L)	134.0 ± 15.4	133.6 ± 20.5	132.5 ± 20.4	0.820	0.537
Platelet (×10^9^/L)	220.5 ± 60.5	225.8 ± 77.5	220.6 ± 70.3	0.909	0.963
ALT (IU/L)	21.5 (14.6/35.2)	22.4 (15.2/36.2)	22.2 (15.5/37.0)	0.695	0.512
AST (IU/L)	21.0 (16.0/35.9)	21.0 (17.2/31.0)	23.2 (17.0/37.1)	0.775	0.983
TBil (umol/L)	11.8 (8.0/16.7)	12.0 (9.0/15.9)	12.0 (8.0/17.7)	0.968	0.357
Albumin (g/L)	39.7 ± 4.8	40.3 ± 5.5	39.1 ± 5.3	0.361	0.300
FBG (mmol/L)	5.1 (4.7/5.9)	5.1 (4.8/6.1)	5.4 (4.7/6.3)	0.100	0.040
eGFR (ml/min)	74.9 ± 27.9	70.2 ± 21.3	72.6 ± 30.8	0.675	0.590
BUN (mmol/L)	5.3 (4.0/7.3)	5.2 (4.1/6.8)	5.4 (4.3/7.0)	0.683	0.857
Uric acid (umol/L)	343.1 ± 111.4	404.3 ± 126.7^*^	383.6 ± 125.1^*^	0.009	0.016
**Natriuretic peptides**					
BNP (pg/mL)	168.5 (120.0/378.4)	164.0 (111.5/466.5)	337 (185.7/649.5)	0.054	0.608
NT-proBNP (pg/mL)	353.5 (100.8/1459.3)	250.0 (110.0/994.2)	1039.0 (235.0/2528.5)^*#^	0.003	0.014
Triglyceride (mmol/L)	1.5 (1.0/2.2)	1.5 (1.0/2.2)	1.4 (1.0/2.0)	0.923	0.827
Cholesterol (mmol/L)	4.6 (3.7/5.5)	4.8 (4.0/5.8)	4.5 (3.6/5.6)	0.409	0.639
LDL (mmol/L)	2.5 (1.7/3.5)	2.9 (1.9/3.3)	2.7 (2.1/3.6)	0.608	0.744
HDL (mmol/L)	1.2 ± 0.4	1.2 ± 0.4	1.1 ± 0.3	0.160	0.092
**RHC**					
mRAP (mmHg)	9.6 ± 3.9	12.7 ± 3.4^*^	14.6 ± 4.2^*#^	<0.001	<0.001
RVSP (mmHg)	28.7 ± 6.2	33.2 ± 7.1^*^	47.8 ± 13.1^*#^	<0.001	<0.001
RVEDP (mmHg)	9.0 (6.8/12.0)	11.0 (9.5/13.0)^*^	15.0 (10.8/16.0)^*#^	<0.001	<0.001
sPAP (mmHg)	28.6 ± 6.9	33.4 ± 4.1^*^	48.2 ± 12.2^*#^	<0.001	<0.001
dPAP (mmHg)	12.9 ± 3.1	18.3 ± 3.1^*^	24.0 ± 7.0^*#^	<0.001	<0.001
mPAP (mmHg)	18.0 (17.0/20.0)	22.0 (21.0/23.0)^*^	30.0 (27.0/36.0)^*#^	<0.001	<0.001
PAWP (mmHg)	17.0 (16.0/20.0)	19.5 (17.0/24.0)	19.0 (17.0/24.5)	0.353	0.581
LVEDP (mmHg)	14.0 (12.0/15.0)	18.0 (16.0/21.0)^*^	17.0 (16.0/20.0)^*^	<0.001	<0.001
**Echocardiography**					
LAAPD (mm)	33.4 ± 5.1	32.8 ± 4.7	36.6 ± 7.2^*#^	<0.001	<0.001
LVEDD (mm)	45.5 ± 6.5	46.1 ± 7.8	50.8 ± 8.5^*#^	<0.001	<0.001
RVAPD (mm)	18.4 ± 3.3	18.4 ± 1.9	20.8 ± 6.1^*#^	<0.001	0.001
LVEF (%)	55.4 ± 10.2	56.6 ± 9.1	53.3 ± 11.0	0.104	0.081
PE, *n* (%)^∫∫^	3 (3.3%)	1 (2.7%)	11 (5.1%)		
**Medications**, ***n*** **(%)**					
Aldactone	25 (27.2%)	11 (29.7%)	125 (58.1%)^*#^	<0.001	<0.001
ACEI	37 (40.2%)	14 (37.8%)	98 (45.6%)	0.532	0.337
ARB	19 (20.7%)	12 (32.4%)	50 (23.3%)	0.321	0.644
Beta blocker	65 (70.7%)	24 (64.9%)	149 (69.3%)	0.811	0.884
Diuretic	27 (29.3%)	9 (24.3%)	108 (50.2%)^*#^	<0.001	<0.001
CCB	16 (17.4%)	6 (16.2%)	44 (20.5%)	0.730	0.491
Statin	83 (90.2%)	31 (83.8%)	185 (86.0%)	0.511	0.370
Antiplatelet	72 (78.3%)	28 (75.7%)	186 (86.5%)	0.092	0.054

Among the 326 patients, the number of missing values for the covariates were: 1 (0.3%) for Platelet; 2 (0.6%) for ALT and Albumin; 3 (0.9%) for AST, Tbil, Uric acid, and RVEDP; 4 (1.2%) for Triglyceride, Cholesterol, HDL, and RVSP; 5 (1.5%) for LDL and mRAP; 6 (1.7%) for FBG; 18 (5.2%) for RVAPD; 42 (12.2%) for Natriuretic peptides.

PH, pulmonary hypertension; mPAP, mean pulmonary artery pressure; BMI, body mass index; NYHA FC, New York Heart Association Functional Class; HF, heart failure; HFrEF, heart failure with reduced ejection fraction; HFmrEF, heart failure with mildly reduced ejection fraction; HFpEF, left ventricular diastolic dysfunction heart failure with preserved ejection fraction; SBP, systolic blood pressure; DBP, diastolic blood pressure; CAD, coronary artery disease; ALT, alanine aminotransferase; AST, aspartate aminotransferase; TBil, total bilirubin; FBG, fasting blood glucose; eGFR, estimated glomerular filtration rate; BUN, blood urea nitrogen; BNP, b-type natriuretic peptide; NT-pro BNP, N-terminal pro b-type natriuretic peptide; LDL, low-density lipoprotein cholesterol; HDL, high-density lipoprotein cholesterol; RHC, right heart catheterization; mRAP; mean right atrial pressure; RVSP, right ventricular systolic pressure; RVEDP, right ventricular end diastolic pressure; sPAP, systolic pulmonary artery pressure; dPAP, diastolic pulmonary artery pressure; mPAP, mean pulmonary artery pressure; PAWP, pulmonary artery wedge pressure; LVEDP, left ventricular end-diastolic pressure; LAAPD, left atrial anteroposterior diameter; LVEDD, left ventricular end diastolic diameter; RVAPD, right ventricular anteroposterior diameter; LVEF, left ventricular ejection fraction; PE, Pericardial effusion; ACEI, angiotensin-converting enzyme inhibitors; ARB, angiotensin receptor blocker; CCB, calcium channel blocker.

* p < 0.05 vs. without PH (mPAP ≤ 20 mmHg).

# p < 0.05 vs. borderline PH (21 ≤ mPAP ≤ 24 mmHg).

∫∫ A Chi-square test was not performed in these variables of very low counts.

### Impact of borderline PH on outcomes

**Figure 2:**
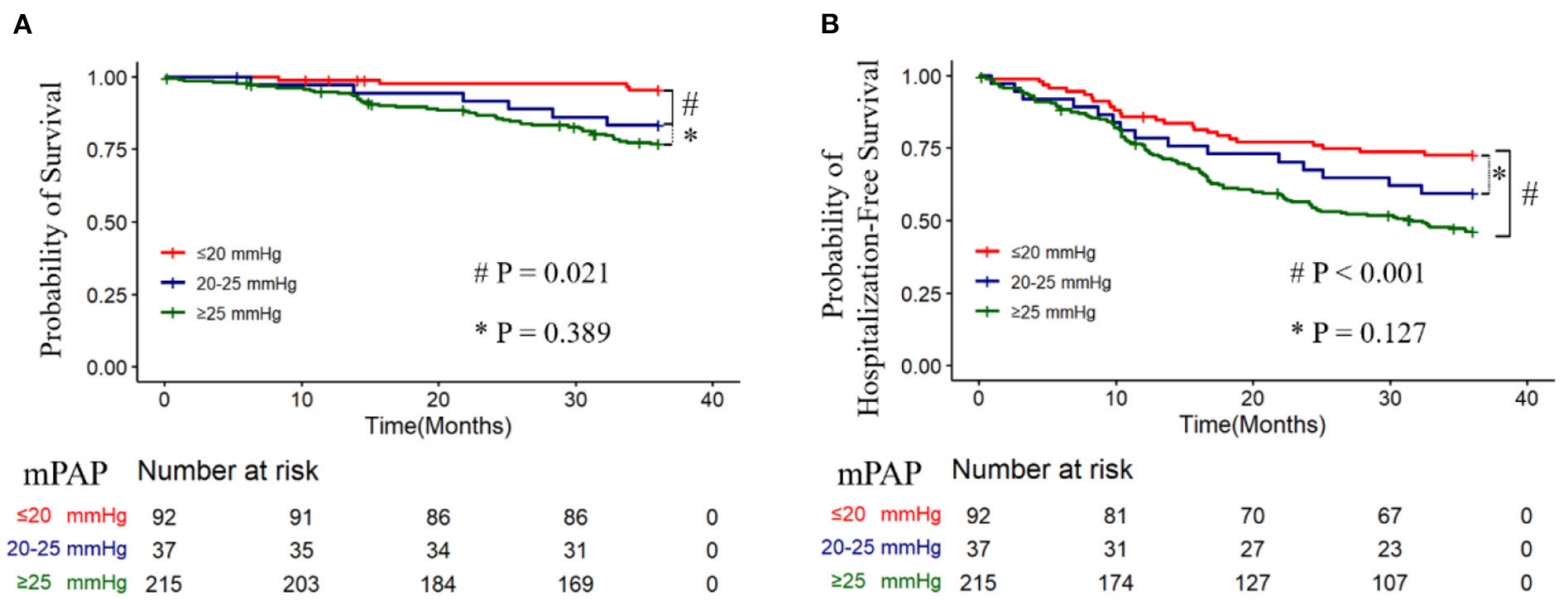
3-year survival and hospitalization-free survival for without pulmonary hypertension (PH) (mPAP ≤ 20 mmHg), borderline PH (21–24 mmHg), and traditionally defined PH (≥25 mmHg) patients, and Kaplan-Meier analysis of the probability of all-cause mortality **(A)** (Log-rank, *p* < 0.001) and rehospitalization **(B)** (Log-rank, *p* < 0.001) was performed.

Within 3 years, 18 (5.2%) patients were lost to follow-up, one with borderline PH, four with non-PH, and 13 with traditionally defined PH. The Kaplan-Meier survival analysis revealed that the unadjusted mortality was intermediated for borderline PH relative to traditionally defined PH and the non-PH group (Figure 2A), with a 3-year mortality rate of 4.5% for the non-PH group, 16.7% for the borderline PH group, and 23.1% for traditionally defined PH group, respectively. The non-PH, borderline PH and traditionally defined PH groups had 1-year death rates of 1.1, 2.8, and 5.2%, respectively, while it was 2.2, 8.3, and 13.3% in 2 years. After controlling for clinical factors that were individually significant in univariate Cox regression analysis, the traditionally defined PH group had the maximum mortality hazard ratio (HR = 4.023; 95% CI: 1.411–11.465; *p* = 0.009). Additionally, a 3.8-fold increase in the risk of adjusted hazard for mortality in the borderline PH group (HR = 3.822; 95% CI: 1.043–13.999; *p* = 0.043) compared to the non-PH group (Table 2) was observed. By employing either forward or backward stepwise regression, borderline and traditionally defined PH were retained in the model and demonstrated the most significant predictive performance. Furthermore, we also observed that the rehospitalization was intermediated for borderline PH relative to traditionally defined PH and the non-PH group (Figure 2B), with 3-year rehospitalization rates of 27.3% for the non-PH group, 40.5% for borderline PH group, and 53.7% for traditionally defined PH group, respectively. The 1-year rehospitalization rates were 14.1% for non-PH, 21.6% for borderline PH, and 24.0% for traditionally defined PH, respectively, while they were 22.9, 32.4, and 43.5%, respectively, after 2 years. The adjusted HR for rehospitalization was highest in patients with traditional defined PH (HR = 2.010; 95% CI: 1.263–3.197; *p* = 0.003), but there was no statistically significant difference in the increased HR in borderline PH patients compared to the non-PH group (HR = 1.599; 95% CI: 0.833–3.067; *p* = 0.158) (Supplement Table S1). The small sample size may explain this result.

**Table 2:** Hazard ratio for mortality among patients assigned to the borderline PH and traditionally defined PH groups compared with the non-PH group.

**Variables**	**Crude HR (95%CI)**	**Crude *P* value**	**Adj. HR (95%CI)**	**Adj. *P* value**
**mPAP**				
Without PH	1.0 (reference)		1.0 (reference)	
Borderline PH	3.970 (1.120–14.069)	0.033	3.822 (1.043–13.999)	0.043
PH	5.760 (2.077–15.975)	0.001	4.023 (1.411–11.465)	0.009
**NYHA FC**				
II	1.0 (reference)		1.0 (reference)	
III/IV	3.658 (2.151–6.220)	<0.001	2.559 (1.392–4.706)	0.002
**Hypertension**				
No	1.0 (reference)		1.0 (reference)	
Yes	2.393 (1.392–4.113)	0.002	2.896 (1.618–5.183)	<0.001
Age (years)	1.044 (1.019–1.070)	<0.001	1.039 (1.005–1.073)	0.024
BUN (mmol/L)	1.140 (1.070–1.214)	<0.001	1.151 (1.059–1.251)	0.001
Uric acid (umol/L)	1.005 (1.003–1.007)	<0.001	1.003 (1.001–1.005)	0.008
NP (BNP ≥288 or				
NT-pro BNP ≥635)				
No	1.0 (reference)			
Yes	1.818 (1.064–3.106)	0.029		
Hemoglobin (g/L)	0.985 (0.974–0.995)	0.003		
FBG (mmol/L)	1.063 (1.002–1.128)	0.042		
eGFR (ml/min)	0.983 (0.973–0.994)	0.002		
HDL (mmol/L)	0.420 (0.188–0.942)	0.035		
LAAPD (mm)	1.044 (1.010–1.079)	0.010		
LVEDD (mm)	1.039 (1.012–1.067)	0.004		

PH, pulmonary hypertension; Adj. HR, adjusted hazard ratio; CI, confidence interval; mPAP, mean pulmonary artery pressure; NYHA FC, New York Heart Association Functional Class; BUN, blood urea nitrogen; NP, Natriuretic peptides; BNP, b-type natriuretic peptide; NT-pro BNP, N-terminal pro b-type natriuretic peptide; FBG, fasting blood glucose; eGFR, estimated glomerular filtration rate; HDL, high-density lipoprotein cholesterol; LAAPD, left atrial anteroposterior diameter; LVEDD, left ventricular end diastolic diameter.

### Impact of mPAP on mortality

**Figure 3:**
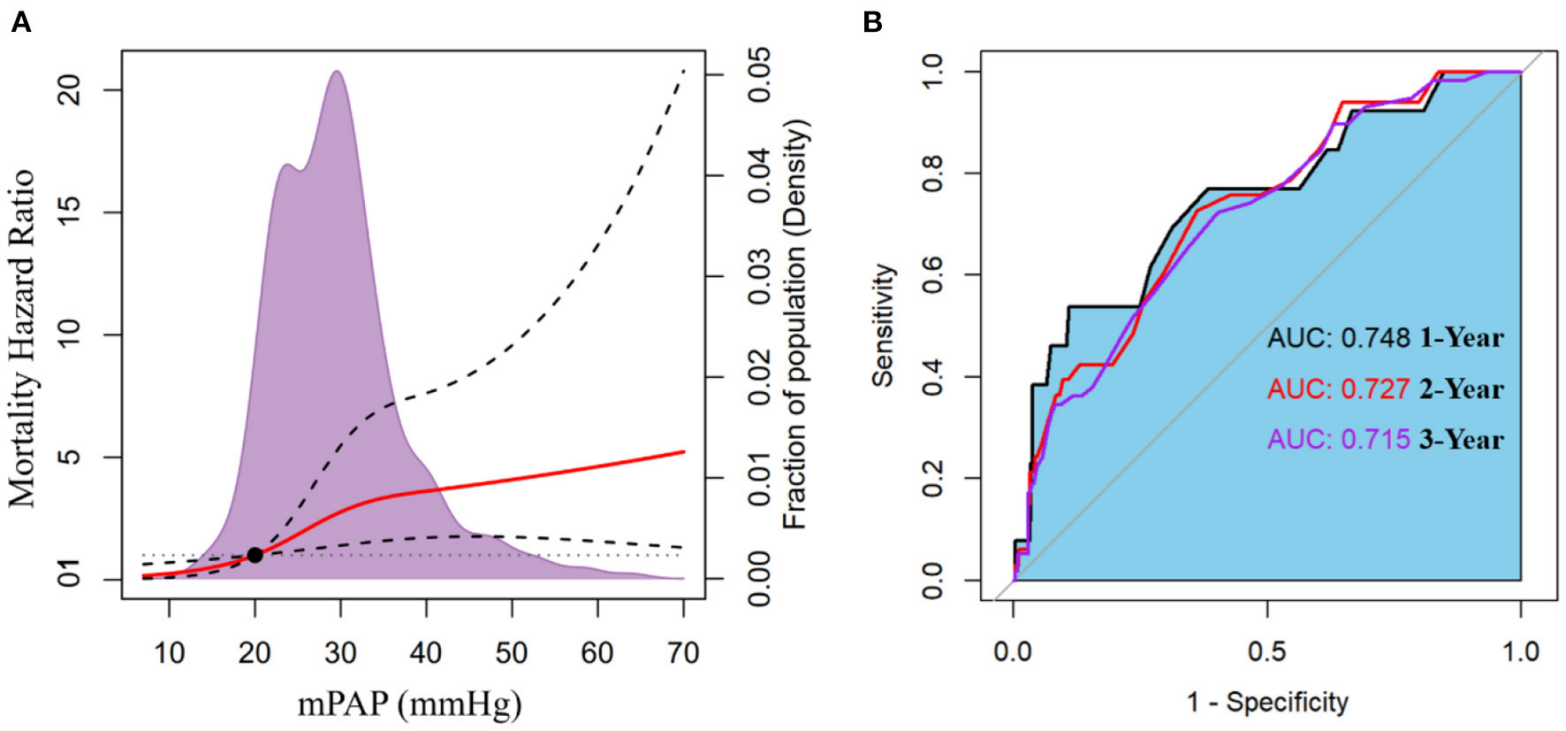
**(A)** The adjusted hazard ratio for mortality according to mean pulmonary artery pressure (mPAP). **(B)** Receiver operator characteristic (ROC) curves with associated area under the curve (AUC) of mPAP to predict mortality at 1-, 2-, and 3-year.

When mPAP was treated as a continuous variable, the adjusted HR for all-cause mortality increased promptly and incrementally across a wide range of mPAP values, beginning at 20 mmHg (HR = 1.006; 95% CI: 1.001–1.012) and continuing to 70 mmHg (Figure 3A). Additionally, it was discovered that a one mmHg incremental increase in mPAP has the most significant effect on death risk between 21 and 24 mmHg, the effect gradually weakened beyond this range (Supplement Figure S2). This finding implies that patients with borderline PH are susceptible when exposed to the progressively increased mPAP. ROC curves indicated that the discriminating power of mPAP to predict death was greatest at 1 year (Figure 3B). ROC analysis identified a cut-off value of 37.5 (mmHg) for mPAP as a predictor of 1-year mortality, with a sensitivity of 53.8%, specificity of 89.1%, positive predictive value (PPV) of 16.3%, and negative predictive value (NPV) of 98.0%.

### Factors associated with new and traditionally defined PH

A logistic regression analysis was used to investigate the association of factors (excluding RHC variables) with new defined PH (mPAP > 20 mmHg) and traditional defined PH (mPAP ≥ 25 mmHg). Multivariate logistic regression analysis showed that left ventricular end diastolic diameter (LVEDD) and right ventricular anteroposterior diameter (RVAPD) were independent predictive factors of both prediction models (for predicting new defined PH, and traditionally defined PH, respectively) (Supplementary Tables S2, S3). LVEDD contributed more predictors to both prediction models than RVAPD, as measured by the partial chi-square statistic minus the predictor degrees of freedom (7.4 vs. 4.3, 10.0 vs. 4.7, respectively).

## Discussion

The current study analysis of LHF patients who underwent RHC from a multicenter registry cohort revealed not just traditionally defined PH but borderline PH is also independently linked with increased 3-year all-cause mortality. Additionally, as the mPAP value was above 20 mmHg, the adjusted mortality hazard ratio increased directly and progressively, with the most remarkable interval change observed in patients with an mPAP between 21 and 24 mmHg. These findings demonstrate that borderline PH has the same clinical significance as traditionally defined PH and supports the new PH hemodynamic criteria (mPAP > 20 mmHg) adopted in LHF patients.

At the first WSPH in 1973, an mPAP > 25 mmHg was used as the hemodynamic threshold for diagnosing PH (13). However, because the conference was focused on primary PH, the threshold of 25 mmHg was intended to differentiate primary PH from other causes of PH, such as left heart disease or chronic lung disease, which typically present with significantly lower mPAP. Therefore, the cut-off value of 25 mmHg was arrived at empirically and pragmatically rather than scientifically. In 2008, the fourth WSPH suggested that an mPAP of <20 mmHg be deemed normal (14). Based on a systematic evaluation of 1,187 individuals from 47 studies, the normal mPAP was reported to be 14.0 mmHg with an SD of 3.3 mmHg. The upper limit of normal (20 mmHg) was determined by adding two SDs (6.6 mmHg) to the mean (14.0 mmHg) (15). As a result, the name “borderline PH” was considered for mPAP values between 21 and 24 mmHg but was rejected due to a lack of management strategies, epidemiology, and prognosis data for this group. Finally, they slightly modified the mPAP value for diagnosing PH (>25 to ≥25 mmHg) (14). Recently, multiple studies indicated an association between modestly raised mPAP and mortality (10, 11, 16, 17). As a result, the 6th WSPH hold in 2018, decided to lower the mPAP threshold to >20 mmHg for all forms of PH (4). However, because none of these studies included subgroup analyses by type of PH, the data on whether borderline PH was related to unfavorable clinical outcomes remained ambiguous across all PH types.

The Veterans Affairs Clinical Assessment, Reporting, and Tracking Program (VA CART) is the most extensive study investigating the relationship between borderline PH and mortality (11). They enrolled 21,727 individuals (23% of borderline PH patients, *n* = 5,030) in the VA CART trial and discovered that the mortality HR rose to start at 19 mmHg (HR = 1.183; 95% CI: 1.004–1.393), and borderline PH is related with increased all-cause mortality and rehospitalization. Our study reveals that the effect of borderline PH on all-cause mortality persists in LHF patients; consequently, we believe that the new definition of PH for LHF patients (mPAP > 20 mmHg) is appropriate. Furthermore, investigations have revealed that borderline PH also has a negative effect on patients' exercise ability, with the 6 min walk distance and peak oxygen uptake significantly lower in borderline PH patients than in the non-PH group (16–18). The repeated RHC data provides the evidence for progression of borderline PH to traditionally defined PH (mPAP ≥25 mmHg), 43 of 70 (61%) patients with borderline PH developed PH that mainly of post-capillary PH, most of whom were undergoing repeated RHC within 1 year (median interval between RHC, 35 weeks; IQR, 12–124 weeks), and the median increase in mPAP was 10 mmHg (10). In this present study, we noticed the proportion of borderline PH was relatively lower, 11% with borderline PH, and 62.0% with traditionally defined PH. Our findings and the repeated RHC data indicate that borderline PH in LHF patients may be a transient stage and relatively rapid transition to PH. The unresolved issue of the two large cohort studies is the causality between borderline PH and mortality. Individuals with borderline PH involved in both studies had a higher prevalence of coronary artery disease, systemic hypertension, diabetes, and heart failure than the non-PH group (10, 11). Although the authors control for cardiac and metabolic disorders, they acknowledge that residual confounding may affect the mortality differences. As a result, it is unknown whether the increased death is due to borderline PH itself or a higher incidence of these illnesses. On the contrary, our study showed no significant differences in these illnesses between the borderline PH and non-PH groups. While this could be because all patients recruited had LHF, our data imply that borderline PH is more likely to cause increased mortality. Experimental and human studies have indicated that borderline PH contributes to right ventricular dysfunction (19, 20). Similarly, our investigation discovered that borderline PH patients had greater right ventricular end-diastolic pressure (RVEDP) than non-PH patients (Table 1). Furthermore, earlier research has established a strong link between right ventricular dysfunction and higher mortality in individuals with LHF (21–23). Consequently, we hypothesize that right ventricular dysfunction may contribute to mortality in borderline PH patients.

In LHF patients, the primary driving force of PH is an increase in left ventricular (LV) filling pressure, which as a result increases left atrial (LA) pressure. Over time, LA loses its ability to act as a buffer zone shielding the pulmonary system from the passive backward transmission of increased LV filling pressure, eventually resulting in PH (24). Therefore, if the underlying LHF continues to deteriorate, the borderline PH will probably develop to the traditionally defined PH. The new definition of PH will enable these individuals (mPAP: 21–24 mmHg) to receive closer monitoring, and intensification of management, perhaps mitigating the longitudinal disease burden.

Current PH guidelines make a weak recommendation for RHC in individuals suspected of having PH due to left heart disease (IIb) (2). For patients with suspected PH-LHF, with no specific PH treatment, early RHC has significant implications for identifying at-risk individuals, as mPAP > 20 mmHg is associated with unfavorable outcomes. Taken together, this observational study established that borderline PH is related to an elevated risk of death from any cause in LHF patients. We hypothesize that patients with PH-LHF may benefit from early diagnosed by RHC, that the future phase of PH-LHF may focus on early diagnosis, and that the role of RHC in patients with suspected PH-LHF justifies further exploration.

## Study limitations

Numerous limitations existed in this study, which should be considered when interpreting our findings. To begin, this was a retrospective analysis of a prospective cohort study, which entails inherent limitations such as selection and referral bias. Second, because the fluid challenge was not included in the protocol for our RHC trial, we may have underestimated the prevalence of PH-LHF by removing patients with PAWP beyond the upper range of normal (13–15 mmHg). Lastly, the two subtypes of PH-LHF (isolated post-capillary PH and combined post-capillary and pre-capillary PH) cannot be separated because the pulmonary vascular resistance parameter is unavailable.

## Conclusions

Borderline PH was an independent predictor of 3-year all-cause mortality in patients with LHF, indicating that borderline PH is clinically significant and supports the revised hemodynamic PH criterion applied in LHF patients. Overall, our findings suggest that the risk of mortality increases immediately and incrementally when the mPAP value exceeds 20 mmHg, and support future prospective studies examining the efficacy of closer interval monitoring or management intensification in patients with borderline PH, as well as the importance of performing RHC early in patients with suspected PH-LHF.

## Data availability statement

The raw data supporting the conclusions of this article will be made available by the authors, without undue reservation.

## Ethics statement

The studies involving human participants were reviewed and approved by the Institutional Review Board of Fuwai Hospital. The patients/participants provided their written informed consent to participate in this study.

## Author contributions

YL and JH contributing to the conception and design. YL drafting the article. LP, SH, JSh, WW, FT, XZ, WS, JSu, and TY data collection, analysis, and interpretation. RQ and HH revising the article. All authors contributed to the article and approved the submitted version.

## Funding

This study was supported by the National Key Technology R&D Program of China (Project No: 2011BAI11B15) and the National Key Research and Development Program of China (Project No: 2016YFC1304400).

## Conflict of interest

The authors declare that the research was conducted in the absence of any commercial or financial relationships that could be construed as a potential conflict of interest. The handling editor JQ declared a shared affiliation with the author(s) JSh at the time of review.

## Publisher's note

All claims expressed in this article are solely those of the authors and do not necessarily represent those of their affiliated organizations, or those of the publisher, the editors and the reviewers. Any product that may be evaluated in this article, or claim that may be made by its manufacturer, is not guaranteed or endorsed by the publisher.
