# Supplementary material for: Impact of borderline pulmonary hypertension due to left heart failure on mortality in a multicenter registry study: A 3-year survivorship analysis
Source: Front Cardiovasc Med. 2022 Aug 12;9:983803. doi: 10.3389/fcvm.2022.983803 (PMC9412236; doi:10.3389/fcvm.2022.983803)
Supplement: Supplementary file 1 [file Data_Sheet_1.DOCX]

**Supplement materials**

**Supplement Figure 1.** Distribution of mean pulmonary artery pressure (mPAP) measured by right heart catheterization for the study cohort. A histogram demonstrating the distribution of mPAP pressure among the cohort of 344 patients.

**Supplement Figure 2.** Incremental increase in hazard of mortality per 1 mmHg increase in mean pulmonary arterial pressure (mPAP), the highest interval change was seen in patients with a mPAP 21 to 24 mmHg.

Supplement Table 1. Hazard ratio for rehospitalization among patients assigned to the borderline PH and traditionally defined PH groups compared with the non-PH group.

| Variables | Crude HR (95%CI) | Crude P value | Adj. HR (95%CI) | Adj. P value |
| --- | --- | --- | --- | --- |
| mPAP |  |  |  |  |
| Without PH | 1.0 (reference) |  | 1.0 (reference) |  |
| Borderline PH | 1.608 (0.848-3.051) | 0.146 | 1.599 (0.833-3.067) | 0.158 |
| PH | 2.331 (1.511-3.597) | <0.001 | 2.010 (1.263-3.197) | 0.003 |
| NYHA FC |  |  |  |  |
| II | 1.0 (reference) |  | 1.0 (reference) |  |
| III/IV | 1.844 (1.340-2.537) | < 0.001 | 1.469 (1.035-2.085) | 0.031 |
| Hypertension |  |  |  |  |
| No | 1.0 (reference) |  | 1.0 (reference) |  |
| Yes | 1.655 (1.202-2.277) | 0.002 | 1.528 (1.093-2.136) | 0.013 |
| Types of HF |  |  |  |  |
| HFpEF | 1.0 (reference) |  |  |  |
| HFmrEF | 1.803 (1.188-2.736) | 0.006 |  |  |
| HFrEF | 1.158 (0.711-1.888) | 0.555 |  |  |
| NP (BNP ≥288 or |  |  |  |  |
| NT-pro BNP ≥635） |  |  |  |  |
| No | 1.0 (reference) |  |  |  |
| Yes | 1.524 (1.106-2.100) | 0.010 |  |  |
| Age (years) | 1.029 (1.014-1.043) | < 0.001 |  |  |
| BUN (mmol/L) | 1.056 (1.004-1.110) | 0.035 |  |  |
| Uric acid (umol/L) | 1.002 (1.000-1.003) | 0.022 |  |  |
| Hemoglobin (g/L) | 0.989 (0.982-0.997) | 0.008 |  |  |
| eGFR (ml/min) | 0.988 (0.982-0.994) | < 0.001 |  |  |
| LVEDD (mm) | 1.021 (1.002-1.039) | 0.028 |  |  |

PH, pulmonary hypertension; CI, confidence interval; Adj. HR, adjusted hazard ratio; mPAP, mean pulmonary artery pressure; NYHA FC, New York Heart Association Functional Class; HF, heart failure; HFrEF, heart failure with reduced ejection fraction; HFmrEF, heart failure with mildly reduced ejection fraction; HFpEF, left ventricular diastolic dysfunction heart failure with preserved ejection fraction; BUN, blood urea nitrogen; eGFR, estimated glomerular filtration rate; NP, natriuretic peptide; LVEDD, left ventricular end diastolic diameter.

* The variable of NP is a combination of BNP and NT-proBNP

Supplement Table 2. Predict factors for new defined PH (mPAP > 20 mmHg) in univariate and multivariate logistic regression analysis.

| Variables | Crude OR (95%CI) | Crude P value | Adj. OR (95%CI) | Adj. P value |
| --- | --- | --- | --- | --- |
| NYHA FC |  |  |  |  |
| II | 1.0 (reference) |  |  |  |
| III/IV | 2.215 (1.269-3.866) | 0.005 |  |  |
| DBP (mmHg) | 0.981 (0.963-0.999) | 0.037 | 0.978 (0.959-0.997) | 0.024 |
| Uric acid (umol/L) | 1.003 (1.001-1.005) | 0.003 |  |  |
| NP (BNP ≥288 or  NT-pro BNP ≥635） |  |  |  |  |
| NO | 1.0 (reference) |  |  |  |
| YES | 1.795 (1.104-2.919) | 0.018 |  |  |
| LAAPD (mm) | 1.078 (1.030-1.130) | 0.001 |  |  |
| LVEDD (mm) | 1.091 (1.049-1.135) | < 0.001 | 1.080 (1.025-1.137) | 0.004 |
| RVAPD (mm) | 1.194 (1.077-1.324) | 0.001 | 1.133 (1.019-1.260) | 0.021 |

PH, pulmonary hypertension; OR, odds ratio; CI, confidence interval; Adj. OR, adjusted odds ratio; NYHA FC, New York Heart Association Functional Class; DBP, diastolic blood pressure; NP, Natriuretic peptides; BNP, b-type natriuretic peptide; NT-pro BNP, N-terminal pro b-type natriuretic peptide; LAAPD, left atrial anteroposterior diameter; LVEDD, left ventricular end diastolic diameter; RVAPD, right ventricular anteroposterior diameter.

Supplement Table 3. Predict factors for traditionally defined PH (mPAP ≥ 25 mmHg) in univariate and multivariate logistic regression analysis.

| Variables | Crude OR (95%CI) | Crude P value | Adj. OR (95%CI) | Adj. P value |
| --- | --- | --- | --- | --- |
| BMI (kg/m^2^)  NYHA FC | 1.150 (1.053-1.257) | 0.002 |  |  |
| II | 1.0 (reference) |  |  |  |
| III/IV | 2.668 (1.613-4.415) | < 0.001 |  |  |
| CAD |  |  |  |  |
| NO | 1.0 (reference) |  |  |  |
| YES | 0.544 (0.314-0.940) | 0.029 |  |  |
| NP (BNP ≥288 or  NT-pro BNP ≥635） |  |  |  |  |
| NO | 1.0 (reference) |  |  |  |
| YES | 1.836 (1.179-2.858) | 0.007 |  |  |
| LAAPD (mm) | 1.103 (1.055-1.153) | < 0.001 |  |  |
| LVEDD (mm) | 1.100 (1.061-1.141) | < 0.001 | 1.088 (1.035-1.144) | 0.001 |
| RVAPD (mm) | 1.229 (1.116-1.353) | < 0.001 | 1.135 (1.023-1.260) | 0.017 |
| LVEF (%) | 0.978 (0.957-0.999) | 0.043 |  |  |

PH, pulmonary hypertension; OR, odds ratio; CI, confidence interval; Adj. OR, adjusted odds ratio; BMI, body mass index; NYHA FC, New York Heart Association Functional Class; CAD, coronary artery disease; NP, Natriuretic peptides; BNP, b-type natriuretic peptide; NT-pro BNP, N-terminal pro b-type natriuretic peptide; LAAPD, left atrial anteroposterior diameter; LVEDD, left ventricular end diastolic diameter; RVAPD, right ventricular anteroposterior diameter; LVEF, left ventricular ejection fraction.


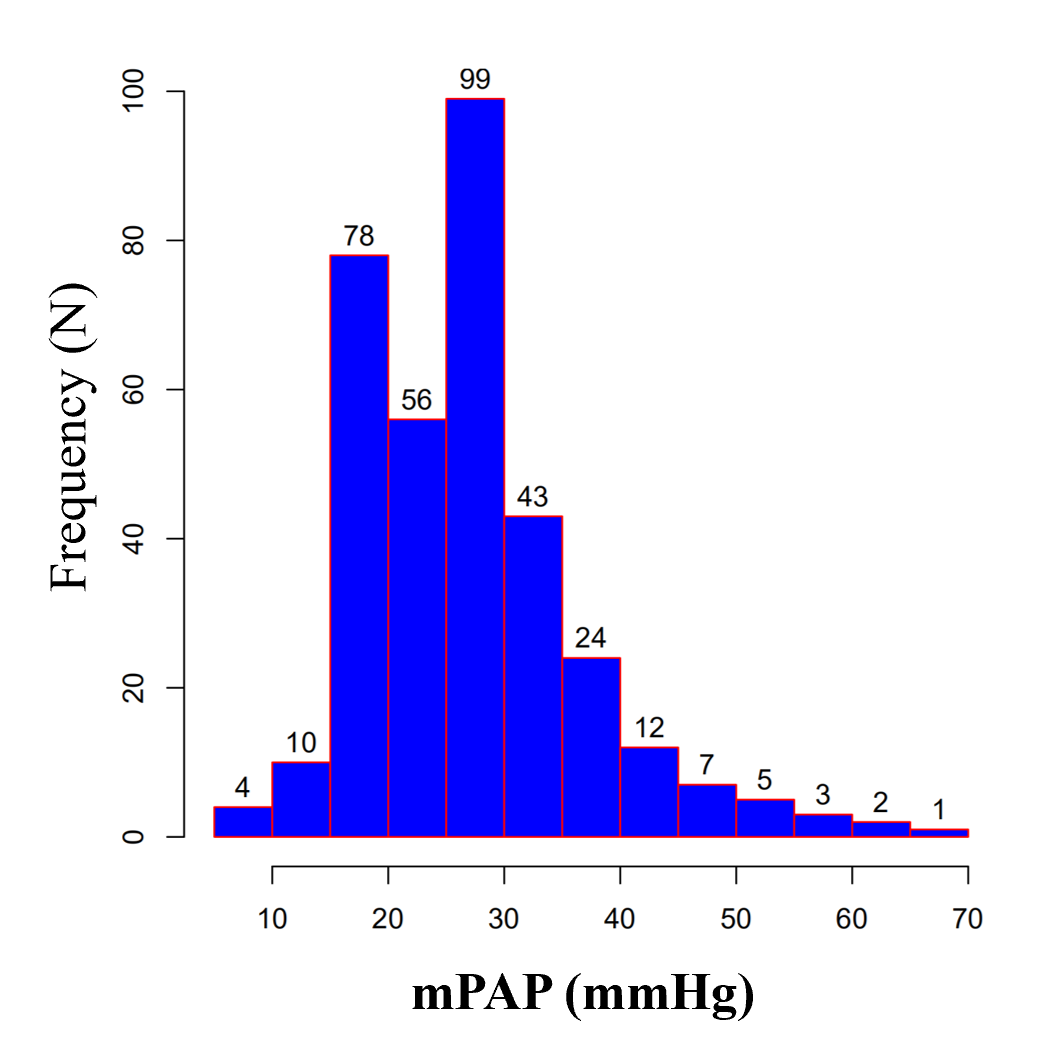


**Supplement Figure 1.** Distribution of mean pulmonary artery pressure (mPAP) measured by right heart catheterization for the study cohort. A histogram demonstrating the distribution of mPAP pressure among the cohort of 344 patients.


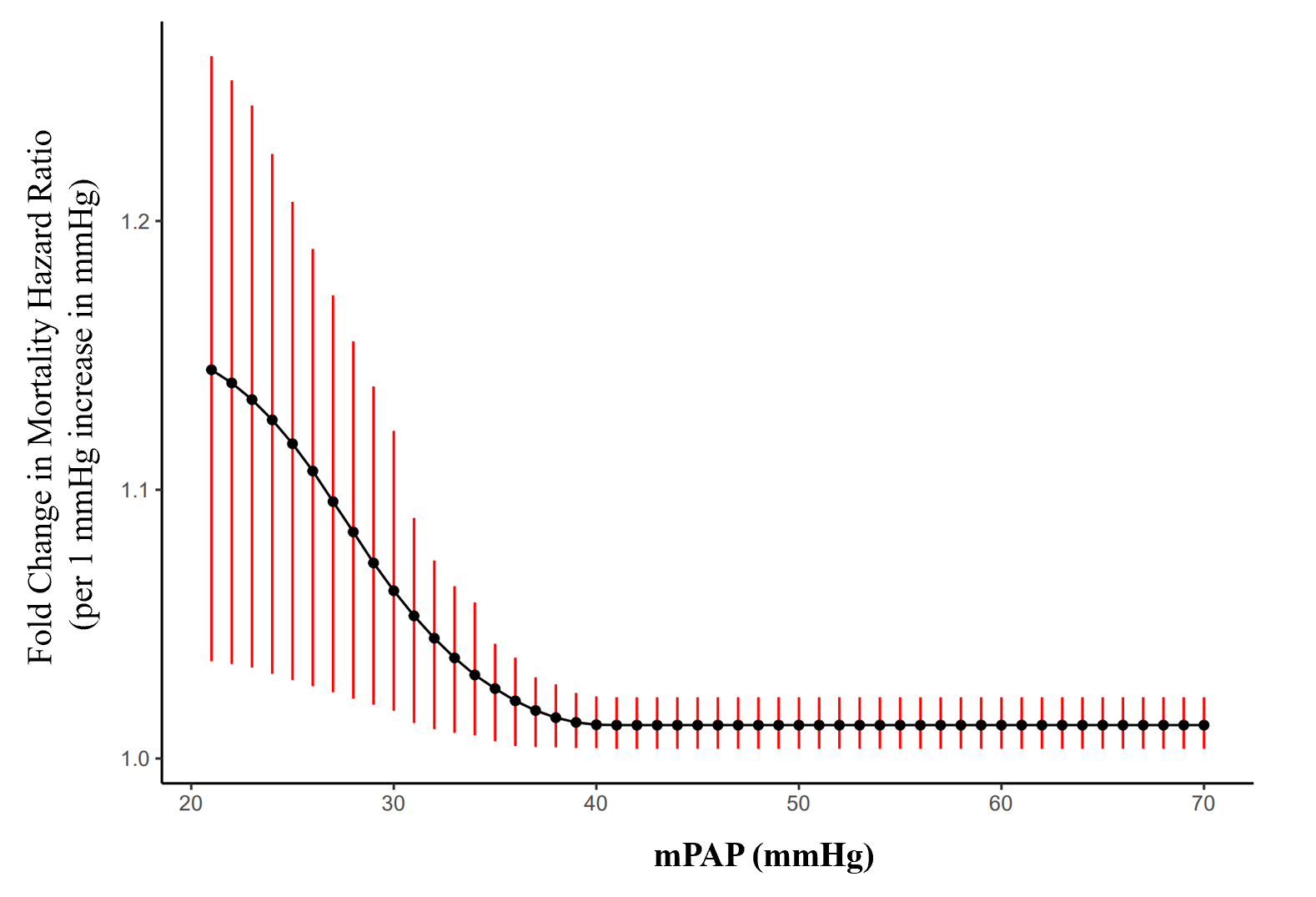


**Supplement Figure 2.** Incremental increase in hazard of mortality per 1 mmHg increase in mean pulmonary arterial pressure (mPAP), the highest interval change was seen in patients with a mPAP 21 to 24 mmHg.
